# Functional Interchangeability of Nucleotide Sugar Transporters URGT1 and URGT2 Reveals That *urgt1* and *urgt2* Cell Wall Chemotypes Depend on Their Spatio-Temporal Expression

**DOI:** 10.3389/fpls.2020.594544

**Published:** 2020-12-08

**Authors:** Jonathan Celiz-Balboa, Asier Largo-Gosens, Juan Pablo Parra-Rojas, Verónica Arenas-Morales, Pablo Sepulveda-Orellana, Hernán Salinas-Grenet, Susana Saez-Aguayo, Ariel Orellana

**Affiliations:** ^1^Centro de Biotecnología Vegetal, Universidad Andrés Bello, Santiago, Chile; ^2^FONDAP Center for Genome Regulation, Facultad de Ciencias de la Vida, Universidad Andrés Bello, Santiago, Chile

**Keywords:** nucleotide sugar, nucleotide sugar transporter, Golgi apparatus, polysaccharides, plant cell wall, pectins, rhamnose, galactose

## Abstract

Nucleotide sugar transporters (NSTs) are Golgi-localized proteins that play a role in polysaccharide biosynthesis by transporting substrates (nucleotide sugars) from the cytosol into the Golgi apparatus. In Arabidopsis, there is an NST subfamily of six members, called URGTs, which transport UDP-rhamnose and UDP-galactose *in vitro*. URGTs are very similar in protein sequences, and among them, URGT1 and URGT2 are highly conserved in protein sequence and also showed very similar kinetic parameters toward UDP-rhamnose and UDP-galactose *in vitro*. Despite the similarity in sequence and *in vitro* function, mutants in *urgt1* led to a specific reduction in galactose in rosette leaves. In contrast, mutants in *urgt2* showed a decrease in rhamnose content in soluble mucilage from seeds. Given these specific and quite different chemotypes, we wonder whether the differences in gene expression could explain the observed differences between the mutants. Toward that end, we analyzed whether URGT2 could rescue the *urgt1* phenotype and *vice versa* by performing a promoter swapping experiment. We analyzed whether the expression of the *URGT2* coding sequence, controlled by the *URGT1* promoter, could rescue the *urgt1* rosette phenotype. A similar strategy was used to determine whether URGT1 could rescue the *urgt2* mucilage phenotype. Expression analysis of the swapped genes, using qRT-PCR, was similar to the native *URGT1* and *URGT2* genes in wild-type plants. To monitor the protein expression of the swapped genes, both URGTs were tagged with green fluorescent protein (GFP). Confocal microscopy analyses of the swapped lines containing URGT2-GFP showed fluorescence in motile dot-like structures in rosette leaves. Swapped lines containing URGT1-GFP showed fluorescence in dot-like structures in the seed coat. Finally, the expression of *URGT2* in *urgt1* mutants rescued galactose reduction in rosette leaves. In the same manner, the expression of *URGT1* in *urgt2* mutants recovered the content of rhamnose in soluble mucilage. Hence, our results showed that their expression in different organs modulates the role *in vivo* of URGT1 and URGT2. Likely, this is due to their presence in different cellular contexts, where other proteins, acting in partnership, may drive their functions toward different pathways.

## Introduction

Nucleotide sugar (NS) transporters (NSTs) are Golgi transmembrane proteins playing a role in the biosynthesis of cell wall matrix polysaccharides (hemicellulose and pectin), which are significant components of plant cell walls (Reyes and Orellana, 2008; Caffall and Mohnen, 2009; Temple et al., 2016). NSTs play important roles in the biosynthesis of non-cellulosic polysaccharides because they transport NSs from the cytosol to the Golgi lumen, where glycosyltransferases (GTs) use them as building blocks to polymerize these polysaccharides (Reyes and Orellana, 2008; Temple et al., 2016). The transport mechanism of NSTs is an antiporter system, in which the transport of one NS into the Golgi lumen results in the transport of one nucleoside monophosphate (NMP) as a counter-exchange molecule from the Golgi to the cytosol (Reyes and Orellana, 2008; Rautengarten et al., 2014, 2017; Ebert et al., 2015; Saez-Aguayo et al., 2017).

In Arabidopsis, 44 genes encode for putative NSTs that form the NST/TPT gene family, which is divided into six clades (Rautengarten et al., 2014). Clade I contains the UDP-rhamnose (UDP-Rha) and UDP-galactose (UDP-Gal) transporters (URGTs), a subfamily composed of six transporters, named URGT1–URGT6, which are able to transport UDP-Rha and UDP-Gal *in vitro* (Rautengarten et al., 2014). Mutations in *URGT1* and *URGT2* led to observable phenotypes associated with defects in pectin composition *in vivo* (Rautengarten et al., 2014; Parra-Rojas et al., 2019). *urgt2* mutants exhibited a reduction in mucilage content with a decrease of rhamnose (Rha) and galacturonic acid (GalA) amounts, which are the basic units of the rhamnogalacturonan I (RG-I) polymer, the main component of the soluble mucilage (SM) layer, suggesting that *URGT2* is involved in the synthesis of the mucilage RG-I polymer (Rautengarten et al., 2014; Takenaka et al., 2018; Parra-Rojas et al., 2019). In contrast to *urgt2*, *urgt1* mutants did not exhibit an SM phenotype but showed a small reduction of galactose (Gal) content in rosette leaves (Rautengarten et al., 2014).

Interestingly, URGT1 and URGT2 protein sequences are very similar, and also, proteins have similar affinity for UDP-Rha and UDP-Gal as well as similarities in their kinetic parameters *in vitro* (Rautengarten et al., 2014). Despite their similarities in protein sequence and in substrate transport affinity *in vitro*, *urgt1* and *urgt2* mutants showed very different cell wall phenotypes. What is the reason for these differences? One explanation could be their gene expression patterns. *URGT1* showed a constitutive expression throughout plant development, while *URGT2* has a specific expression in flowers and seed coat integument (Rautengarten et al., 2014). Moreover, although *URGT1* and *URGT2* have similar expressions in developing seeds, the eFP Browser data revealed that the expression of *URGT2* is more abundant in the seed coat integument in the linear cotyledon seed developmental stage (Supplementary Figures 4, 5). This possibility opens an interesting avenue to explore regarding the manner in which NSTs, which transport more than a single NS, may play their role *in vivo.* Perhaps the most likely explanation for a change in the monosaccharide content of a polysaccharide due to the lack of an NST could be the reduction of NS pools in the Golgi lumen, and thus this could be the limiting factor in the synthesis of certain polysaccharides. However, another interesting possibility is that the activity of an NST could be modulated by its expression in different cell types; thus, depending on the presence of a certain set of proteins that could physically interact with the transporter, different metabolic pathways could be favored; therefore, the phenotype observed in a mutant also depends on the cellular context where the NST is being expressed. To address this question, we conducted a promoter swap experiment of *URGT1* and *URGT2* to determine whether one gene could rescue the phenotype produced by the lack of the other gene. Using this approach, we examined if the expression in rosette leaves of *URGT2* under the control of *URGT1* promoter could rescue the *urgt1* phenotype. In the same way, we explored whether expression of *URGT1* under the control of the *URGT2* promoter in developing seeds could rescue the *urgt2* SM phenotype. Our results show that promoter swap transgenic lines successfully rescue the biochemical changes observed in mucilage and rosette leaves from *urgt2* and *urgt1*, respectively, supporting the hypothesis that these two proteins have similar function and that the Spatio-temporal expressions of *URGT1* and *URGT2* are essential to determine the specific role of each transporter *in vivo*.

## Materials and Methods

### Plant Material and Growth Conditions

Seeds from the Arabidopsis wild-type (WT) Col-0 ecotype and the T-DNA insertional mutant lines *urgt1-2* (SAIL_768_C08) and *urgt2-2* (SALK_071647) were obtained from the ABRC^1^ using the SIGnAL Salk collection (Alonso et al., 2003). Plants were germinated and grown on soil (complete mix of top crop substrate) in a growth chamber under long-day conditions (photoperiod of 16 h light at 21°C and 8 h dark at 18°C, 65% relative humidity, and 170 μmol m^–2^ s^–1^). In all comparative analysis seeds from WT, mutants and transgenic plants had been simultaneously cultivated and harvested.

### Sequence Alignment

URGT protein sequences were obtained from The Arabidopsis Information Resource (TAIR) database^2^ (Lamesch et al., 2012). Full-length amino acid sequences from URGT1/AT1G76670, URGT2/AT2G21070, URGT3/AT5G42420, URGT4/AT4G39390, URGT5/AT4G09810, and URGT6/AT1g34020 were aligned using Multiple Sequence Comparison by Log-Expectation (MUSCLE)^3^, and percentages of identity and similarity were calculated with the Sequence Manipulation Suite^4^.

#### Cloning, Plant Transformation, and Transgenic Plant Selection

The intergenic region of 2,025 bp between the upstream *URGT1* (At1g76680) gene and At1g76670/*URGT1* gene and the intergenic region of 778 bp between At1g21080 and At1g21070/*URGT2* genes were defined as the *URGT1* (proURGT1) and *URGT2* (proURGT2) promoter regions, respectively. Those regions were amplified by PCR from Arabidopsis genomic DNA extracted from leaves using the following primers: proURGT1Fw 5′-AT CACTTCTTTTATTTGGTTT-3′, proURGT1Rev 5′-TTGGATT TGAGAAAATTGAAC-3′, proURGT2Fw 5′-TCATGTGTTG CGAATCTTATTC-3′, and proURGT2Rev 5′-TTGGATTC AAATTAAAAAAATTCGAAATCTGAAATC-3′. PCR products were purified and inserted into the pENTR^TM^/5′-TOPO^®^ cloning vector according to the standard protocol (Thermo Fisher Scientific) to generate the pENTR5-proURGT1 and pENTR5-proURGT2 entry vectors. In parallel, the coding sequence for each URGT was cloned from cDNA synthesized from RNA extracted from Arabidopsis leaves. Sequences without native stop codon were PCR-amplified using the following primer: FwURGT1 5′-CACCATGGAGAAACCGGAGAGCGAG-3′, RevURGT1 5′-TGGTTTAGTGTCACCGAGTTCA-3′, FwURGT2 5′-CACCATGGAGAAAGCAGAGAACGAGA-3′, and RevURGT2 5′-TGCTTTATTATTTCCAAGCTCCAT-3′. PCR products were introduced into the pENTR^TM^/D-TOPO^®^ cloning vector (Thermo Fisher Scientific) to generate the pENTR-URGT1 and pENTR-URGT2 entry vectors. The green fluorescent protein (GFP) sequence was amplified from the pSITE-2CA vector using primers GFPFw 5′-GG GGACAGCTTTCTTGTACAAAGTGGCGATGGTGAGCAAG GGCGAGGAG-3′ and GFPRev 5′-GGGGACAACTTT GTATAATAAAGTTGGTTACTTGTACAGCTCGTCCATGC-3′. This was inserted into pDONR^TM^ P2r-P3 vector through BP reaction (Invitrogen, Life Technologies), to obtain the entry vector p2RP3-GFP. To generate the final expression vectors, each entry vector containing the *URGT* promoter region, the coding sequence, and the GFP sequence was recombined into the pH7m34GW destination vector (Karimi et al., 2005), using the Multisite Gateway technology (Invitrogen, Life Technologies).

After sequence verification of all constructs by sequencing, selected constructs were transferred into the *Agrobacterium tumefaciens* strain GV3101 (Holsters et al., 1978), and then, they were transformed into *urgt1-2* and *urgt2-2* mutant lines by the floral dip method (Clough and Bent, 1998). All the constructs and transgenic lines are illustrated in Figure 2.

For transgenic line selection, screening of the T2 generation plants was carried out by segregation analysis (3:1) on hygromycin. Chi-square analysis for transgene inheritance was performed to analyze the segregation and select transgenic lines with one insertion in their genome. Subsequently, the identification of homozygous transgenic plants was carried out by segregation with a total hygromycin resistance in the T3 generation plants. Three independent transgenic lines were selected for each genotype.

### Quantitative Real-Time PCR Analysis

Total RNA was extracted from 6-week-old rosette leaf tissue and from 8-DAP developing seeds using TRIzol reagent (Thermo Fisher Scientific, United States) and RNeasy Plus Micro Kit (Qiagen), respectively. RNA quality and integrity were determined using the Epoch^TM^ Microplate Spectrophotometer and agarose gel electrophoresis. RNA was treated with 1 μl of DNase I (Thermo Fisher Scientific, United States) and reverse transcribed with SuperScript II (Thermo Fisher Scientific) according to the manufacturer’s instructions. The qRT-PCR assay was performed in a 10-μl final reaction mixture according to the instructions for Fast EvaGreen^®^ qPCR Master Mix (Biotium, United States) using the Stratagene Mx3000P real-time PCR system. The quantification and normalization procedures were done using the equation described in Saez-Aguayo et al. (2017). *EF1aA4* (North et al., 2007), *Clathrin adaptor complex subunit* (AT5G46630), and *Seed reference gene* (At4g12590) (Hong et al., 2010) were used as reference genes, and all primers used in this study were described in Saez-Aguayo et al. (2017) and Parra-Rojas et al. (2019). The reaction was performed on RNA extracted from three biological replicates and was analyzed via qRT-PCR with three technical replicates each.

### Confocal Microscopy Analysis

To stain the cell walls, 8-DAP developing seeds and 6-week-old leaves from transgenic lines were immersed in 20 μM propidium iodide solution for 10 min. After staining, seeds and leaves were rinsed three times with 1× TBS, mounted in water, and observed using a Leica TCS SP8 confocal laser scanning microscope using a 60× objective with 4× digital zoom. The visualization of the GFP fluorescence was performed with laser excitation at 488 nm for GFP fluorescence and 543 nm for propidium iodide. The emission signal was collected between 500 and 570 nm for GFP and between 550 and 725 nm for propidium iodide. Developing seeds and leaves, at the same stage of development, from WT untransformed plants were also examined as negative controls.

### Cell Wall Monosaccharide Composition Analysis

#### Extraction of SM and Alcohol-Insoluble Residue Preparation From Leaves

To extract the SM layer of the WT, *urgt1-2* and *urgt2-2* mutants, and transgenic lines, 50 mg of dry seeds was imbibed three times with 4 ml of water for 30 min at RT. Supernatants were recovered after centrifugation at 8,000 × *g* for 5 min and pooled to obtain the SM fraction. The SM fractions were later lyophilized, resuspended in 300 μl of deionized water, and stored at −20°C until monosaccharide analysis.

To prepare alcohol-insoluble residue (AIR), 6-week-old rosette leaves were ground under liquid nitrogen and then washed overnight with 80% ethanol. The supernatant was removed after centrifugation at 10,000 × *g* for 5 min. The residue was washed three times with 80% ethanol [1 h at room temperature (RT)]. Lipids were extracted by three incubations with methanol/chloroform 1/1, *v*/*v* (1 h, RT) with subsequent centrifugation, and the pellet was washed two times with 100% acetone (1 h, RT). The pellet was collected by centrifugation at 10,000 × *g*, and the obtained AIR was dried overnight at RT.

#### Trifluoroacetic Acid Hydrolysis and Monosaccharide Composition Analysis by HPAEC-PAD

Soluble mucilage fractions (50 μl) and AIR from leaves (1.5 mg) were hydrolyzed with 400 μl of 2 *N*-trifluoroacetic acid (TFA) at 121°C for 45 min. TFA was evaporated at 65°C under nitrogen gas, and the samples were washed twice with 400 μl of 100% isopropanol and dried with nitrogen gas. Hydrolyzed products were resuspended in 700 μl of water and sonicated for 15 min using an Ultrasonic Cleaner (VWR International, United States). Samples were filtered by passing through a syringe filter (pore size: 0.45 mm) and transferred to a new tube. High-performance anion exchange chromatography with pulsed amperometric detection (HPAEC-PAD, Dionex ICS-3000) was performed to analyze the monosaccharide content of each sample, according to Saez-Aguayo et al. (2017). Myo-inositol and allose were used as internal standard for TFA hydrolysis. The analysis was performed using three biological repeats with four technical replicates.

### Statistical Analysis

At least three independent cultures were used to perform each experiment. Statistical analyses were performed by comparison of mutants and transgenic lines with WT using a non-parametric Mann–Whitney test. Data analysis was performed with Prism 6 application (GraphPad software).

## Results

### *URGT1* and *URGT2* Encode Very Similar Protein Sequences but Their Lack of Function Lead to Different Cell Wall Chemotypes

**FIGURE 1:**
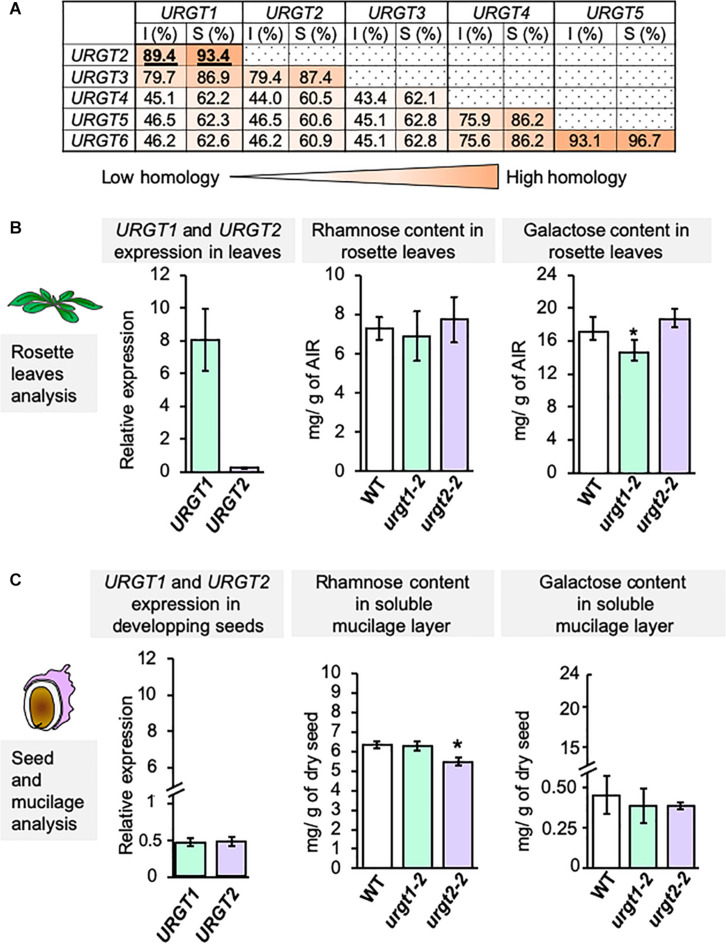
URGT1 and URGT2 have similar protein sequences, but their mutants exhibited specific cell wall phenotypes. **(A)** URGT1 and URGT2 are very similar proteins. Identity (I) and similarity (S) percentages of URGT family protein sequences. URGT proteins were aligned with the MUSCLE tool (https://www.ebi.ac.uk/Tools/msa/muscle/), and percentages were calculated with the Sequence Manipulation Suite (http://www.bioinformatics.org/sms2/ident_sim.html). **(B)** Level of *URGT1* and *URGT2* gene expression in WT leaves and the effect of their mutation in rosette leaf chemotype. Left graph: Analysis of *URGT1* and *URGT2* expression in WT rosette leaves. qRT-PCR analyses of *URGT1* and *URGT2* transcripts were performed in 6-week-old rosette leaves from WT plants. The values were calculated relative to *Clathrin* (*At5g46630*) and *Ef1*α*A4* gene. Error bars represent SE values from three biological replicates (*n* = 9). Middle and right graphs: Analysis of rhamnose (middle) and galactose (right) contents in rosette leaves from 6-week-old plants from *urgt1-1* and *urgt2-2* mutant lines. Error bars represent SD values from three biological replicates (*n* = 12). Statistical analyses were performed by using Mann–Whitney test with *p < 0.05. **(C)** Level of *URGT1* and *URGT2* gene expressions in developing seeds and the effect of their mutation in SM chemotype. Left graph: Analysis of *URGT1* and *URGT2* expressions in the WT developing seeds. qRT-PCR analyses of *URGT1* and *URGT2* transcripts were performed in 8-DAP developing seeds from WT plants. The values were calculated relative to *Ef1*α*A4* and the seed-specific reference gene (*At4g12590*). Error bars represent SE values from three biological replicates (*n* = 9). Middle and right graphs: Analysis of rhamnose (middle) and galactose (right) content in the SM layer from mature dry seeds from *urgt1-1* and *urgt2-2* mutant lines. Error bars represent SD values from three biological replicates (*n* = 12). Statistical analyses were performed by using the Mann–Whitney test with *p < 0.05.

Protein sequence similarity analyses among the six members of the URGT family revealed that all members are quite conserved (Figure 1A). Indeed, URGT proteins have a range of identities from 43.1 to 93.1% and sequence similarities from 60.5 to 96.7% (Figure 1A). URGT1 and URGT2 have highly conserved protein sequences (89.4% identity and 93.4% similarity; Figure 1A and Supplementary Figure 1); however, the mutants on each of these genes lead to different phenotypes (Rautengarten et al., 2014; Parra-Rojas et al., 2019). Indeed, *urgt1* mutant plants show a decrease in the content of galactose in rosette leaves, whereas the content of rhamnose remains invariable. On the other hand, *urgt2* mutant plants exhibit a decrease in the amount of rhamnose, but not in galactose, in SM from seeds (Figures 1B,C). qRT-PCR analyses showed similar levels of expression of both *URGT1* and *URGT2* in developing seeds at 8-DAPs (Figure 1C); however, the expression levels of *URGT1* were notably higher than those of *URGT2* in rosette leaves of 6-week-old plants. It is interesting to note that, although *URGT1* and *URGT2* have similar expression levels in developing seeds, *urgt1* mutants did not show changes in the content of rhamnose and galactose in SM (Figure 1C). Overall, these results suggest that URGT2 contributes to the synthesis of Rha-containing polymers, such as RG-I from seed SM, whereas URGT1 contributes to the synthesis of Gal-containing polymers in rosette leaves.

### *URGT1* and *URGT2* Promoter Swapping as a Manner to Elucidate the *urgt1* and *urgt2* Phenotypes

**FIGURE 2:**
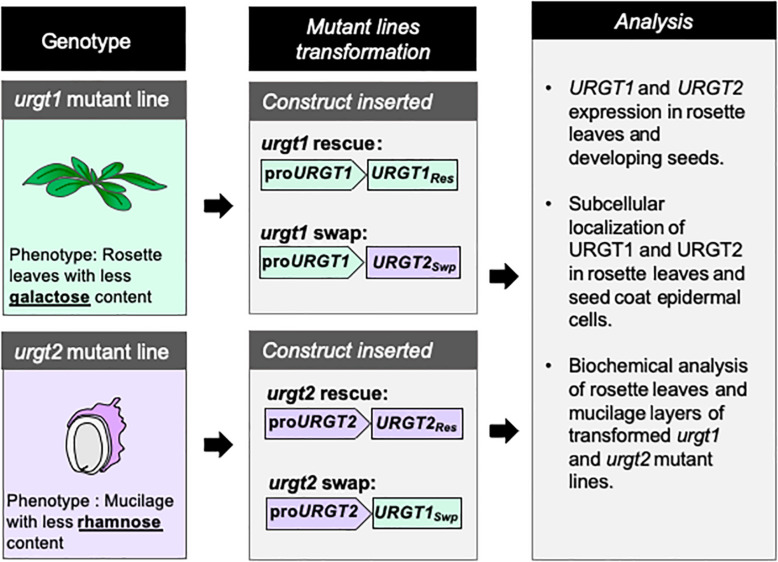
Schematic representation of the strategy used to perform the molecular rescue and promoter swapping of *urgt1* and *urgt2* mutant lines. *urgt1-1* and *urgt2-2* mutant lines were transformed with different constructs. Vectors with _*pro*_URGT1:URGT1-GFP (_*pro*_URGT1:URGT1_*Res*_) and _*pro*_URGT2:URGT2-GFP (_*pro*_URGT2:URGT2_*Res*_) constructs were used to rescue *urgt1-1* and *urgt2-2* mutant phenotypes, respectively. To analyze the effect of the promoter swapping, the constructs _*pro*_URGT1:URGT2-GFP (_*pro*_URGT1:URGT2_*Swp*_) and _*pro*_URGT2:URGT1-GFP (_*pro*_URGT1:URGT2_*Swp*_) were used to transform *urgt1-1* and *urgt2-2*, respectively. To evaluate the effect of the promoter swapping in the transformed *urgt1-1* and *urgt2-2* mutant lines, qRT-PCR, subcellular localization, and biochemical analysis were performed on selected transgenic lines.

Because URGT1 and URGT2 showed similar transport kinetics toward UDP-Rha and UDP-Gal *in vitro* (Rautengarten et al., 2014), we wondered if these two proteins could be exchangeable in their role, providing UDP-Rha and UDP-Gal for the synthesis of RG-I and galactan-enriched pectins. To this end, we used a promoter swapping approach (Muto et al., 2007); thus, the *URGT1* and *URGT2* promoters were exchanged such that the *URGT1* promoter controlled the *URGT2* coding sequence and *vice versa* (Figure 2). As controls, *URGT1* and *URGT2*, under the control of their own promoters, were used to rescue the mutants. Molecular rescued transgenic lines were obtained by transforming the *urgt1-2* and *urgt2-2* mutants with their respective WT genes tagged with GFP as described in Figure 2. To obtain the promoter swapping transgenic lines, the *urgt1-2* mutant was transformed with the construct containing proURGT1:URGT2-GFP (_*pro*_URGT1:URGT2_*Swp*_) and the *urgt2-2* mutant with the construct containing proURGT2:URGT1-GFP (_*pro*_URGT2:URGT1_*Swp*_; Figure 2).

### Effect of Promoter Swapping in the Expression of *URGT1* and *URGT2*

**FIGURE 3:**
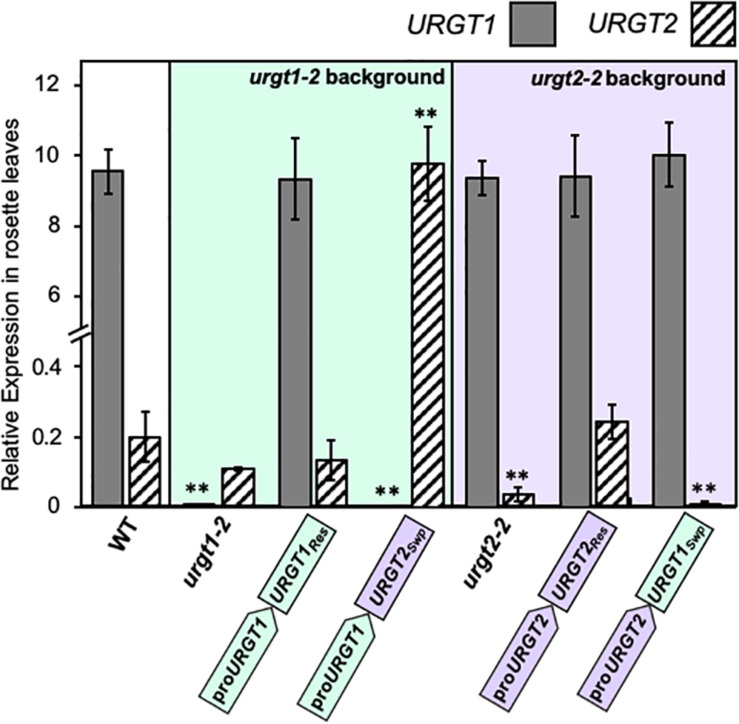
*URGT1* and *URGT2* expressions in 6-week-old rosette leaves of *urgt1* and *urgt2* simple mutants, rescue lines, and promoter swap transgenic lines. qRT-PCR analyses of *URGT1* and *URGT2* transcripts were performed for 6-week-old rosette leaves from *urgt1-2* and *urgt2-2* simple mutants, rescue lines (proURGT1:URGT1 L83 for the *urgt1* mutant and proURGT2:URGT2 L4 for the *urgt2* mutant), and promoter swap transgenic lines (proURGT1:URGT2 L3 for the *urgt1* mutant and proURGT2:URGT1 L3 for the *urgt2* mutant). The values were calculated relative to *Clathrin* (*At5g46630*) and *Ef1aA4* genes. Error bars represent SE values from three biological replicates (*n* = 9). Statistical analyses were performed by using the Mann–Whitney test with **p < 0.001.

**FIGURE 4:**
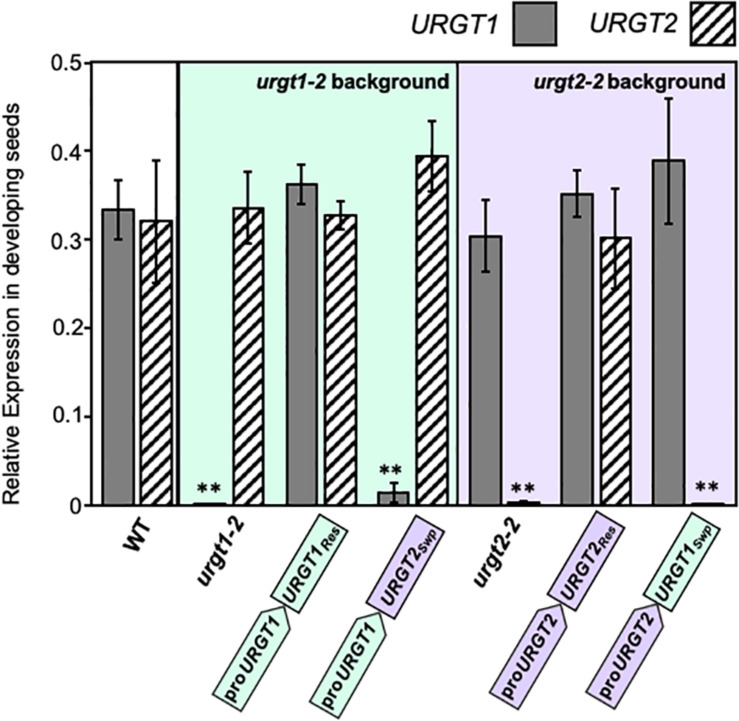
*URGT1* and *URGT2* expressions in 8-DAP developing seeds of *urgt1* and *urgt2* simple mutants, rescue lines, and promoter swap transgenic lines. qRT-PCR analyses of *URGT1* and *URGT2* transcripts were performed in 8-DAP developing seeds from *urgt1-2* and *urgt2-2* simple mutants, rescue lines (proURGT1:URGT1 L83 for the *urgt1* mutant and proURGT2:URGT2 L4 for the *urgt2* mutant), and promoter swap transgenic lines (proURGT1:URGT2 L3 for *urgt1* mutant and proURGT2:URGT1 L3 for *urgt2* mutant). The values were calculated relative to seed-specific reference gene (*At4g12590*) and *Ef1*α*A4* gene expressions. Error bars represent SE values from three biological replicates (*n* = 9). Statistical analyses were performed by using the Mann–Whitney test with **p < 0.001.

As we show below, the WT-GFP-tagged version of the transporters complemented the phenotypes. This bolsters the case that constructs used were functional. To evaluate the expression of each transgene, we analyzed by qRT-PCR the transcript accumulation of *URGT1* and *URGT2* in 6-week-old rosette leaves and 8-DAP developing seeds of WT, *urgt1-2* and *urgt2-2* mutants, and all transgenic lines (Figures 3, 4). Analysis of *URGT1* transcript abundance in the *urgt1-2* mutant showed an almost complete depletion of this transcript in rosette leaves (Figure 3) and developing seeds (Figure 4). The transcript abundance of *URGT2* was not significantly changed in *urgt1-2* (Figures 3, 4 and Supplementary Figures 2, 3). Expression analysis of the *urgt1* rescued transgenic lines (_*pro*_URGT1:URGT1_*Res*_) showed similar levels of the *URGT1* transcript abundance in comparison to WT levels in both tissues, while *URGT2* expression levels remained unchanged. Analyses of *urgt1-2* transformed with _*pro*_URGT1:URGT2_*Swp*_ showed no expression of *URGT1* in 6-week-old rosette leaves; instead, *URGT2* transcript accumulation was significantly higher and reached similar levels to that of WT *URGT1* (Figure 3 and Supplementary Figure 2). The analysis of *URGT1* transcript levels in 8-DAP developing seeds in the same transgenic lines showed a minor increase in the *URGT1* transcript levels (Figure 4 and Supplementary Figure 3), whereas the levels of *URGT2* were not significantly higher than those of WT in the transgenic lines transformed with _*pro*_URGT1:URGT2_*Swp*_. The analyses of the rescued and swapped *urgt2* transgenic lines showed that *URGT2* transcript levels in both 6-week-old rosette leaves and 8-DAP developing seeds reached WT levels in the rescued lines, and no effect of the levels of *URGT1* were observed (Figures 3, 4 and Supplementary Figures 2, 3). On the other hand, the *urgt2-2* promoter swapping lines (_*pro*_URGT2:URGT1_*Swp*_) did not recover the *URGT2* gene expression, and the *URGT1* expression was similar to WT in both tissues (Figures 3, 4 and Supplementary Figures 2, 3).

### Effect of Promoter Swapping on the Localization of URGT1 and URGT2 Proteins

**FIGURE 5:**
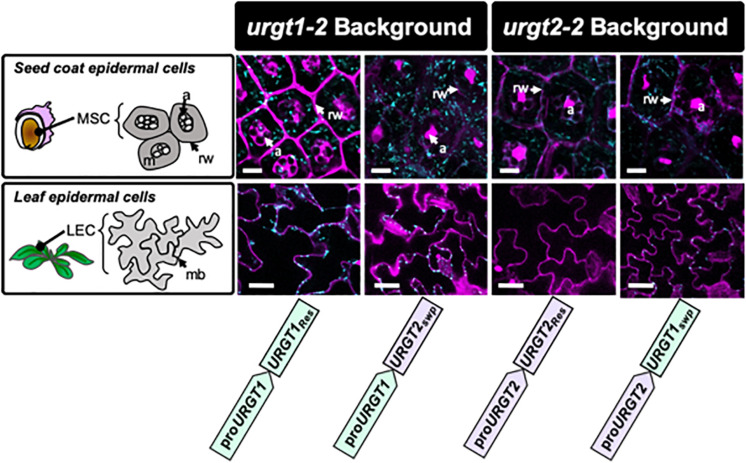
URGT1 and URGT2 subcellular localization in rescue and in promoter swap transgenic lines. Subcellular GFP localization on URGT rescue and promoter swap lines. Confocal laser scanning microscope images of mucilage secretory cells (MSCs) from seed coat of developing seeds at 8 DAP (first row) and leaf epidermal cells (LECs) of 6-week-old rosette leaves (second row). All the images showed that GFP punctuated expression characteristic of Golgi-localized URGTs. Cyan, GFP; Magenta, propidium iodide. MSC, mucilage secretory cell; m, mucilage; rw, radial wall; a, amyloplast; LEC, leaf epidermal cell; mb, membrane (bar = 10 μm).

To evaluate the effect of the promoter swapping strategy on protein localization in seed coat epidermal cells and in rosette leaves, analyses of URGT1-GFP and URGT2-GFP localization were performed in all transgenic lines. Results in Figure 5 showed the presence of fluorescence in seed coat epidermal cells of 8-DAP developing seeds, indicating the presence of URGT1 and URGT2 protein in all rescue and promoter swap transgenic lines. However, the GFP accumulation analysis in rosette leaves revealed the presence of fluorescence in the *urgt1-2* rescued line _*pro*_URGT1:URGT1_*Res*_ and in the leaves from the promoter swap transgenic line _*pro*_URGT1:URGT2_*Swp*_. This result suggested that URGT2-GFP under the control of the URGT1 promoter produces a stable protein in 6-week-old rosette leaves. In contrast, no GFP fluorescence was detected in the *urgt2-2*_*pro*_URGT2:URGT2_*Res*_ rescued line. Furthermore, low GFP fluorescence was observed in rosette leaves from the *urgt2-2*_*pro*_URGT2:URGT1_*Swp*_ swapped line. Based on the higher content of *URGT1* transcripts in comparison to *URGT2* transcripts in this organ (Figure 1), the absence or low levels of URGT2-GFP protein fluorescence could be explained by a possible lower activity of the *URGT2* promoter in comparison to the *URGT1* promoter in rosette leaves. Interestingly, in all the cases where fluorescence was observed, this was localized in motile dot-like structures, a distribution previously reported for both transporters that is consistent with their localization in the Golgi apparatus (Rautengarten et al., 2014).

### The Promoter Swapping Lines Recover the Normal Phenotype in Both *urgt1-2* and *urgt2-2* Plants

**FIGURE 6:**
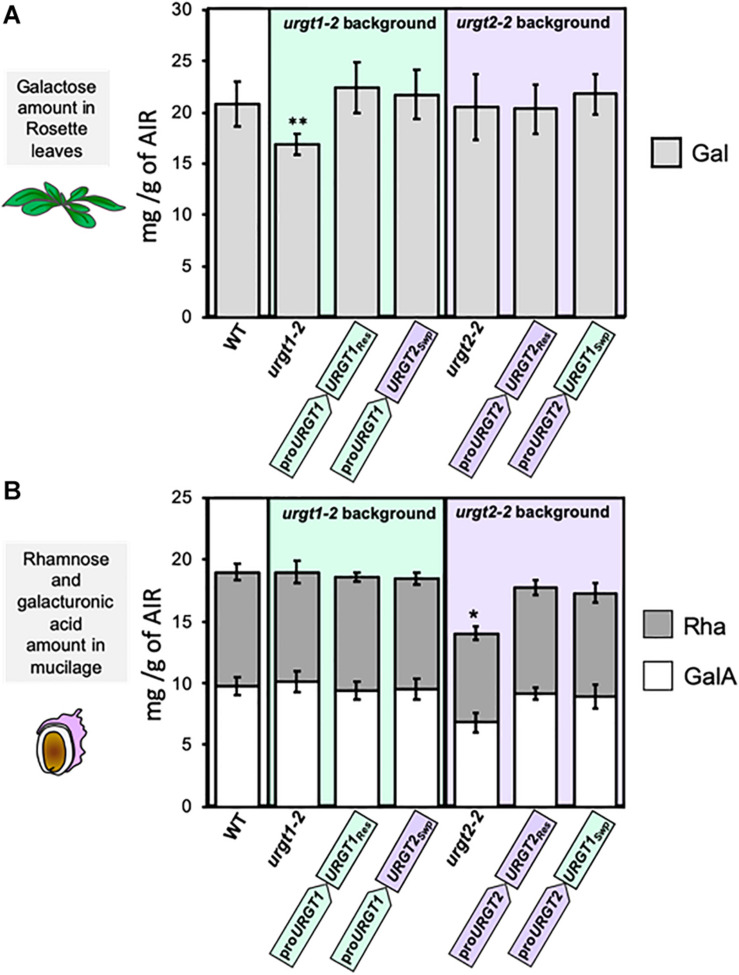
Recovery of *urgt1* and *urgt2* mutant cell wall changes by rescue and promoter swap lines. **(A)** Galactose content in rosette leaves of *urgt1-2* and *urgt2-2* mutants and rescue and promoter swap transgenic lines. Galactose (Gal) amount from AIR of 6-week-old rosette leaves was quantified using HPAEC-PAD. Error bars represent SD from three biological replicates (*n* = 12). Statistical analyses were performed by using the Mann–Whitney test (*p* < 0.05). **(B)** Rhamnose and galacturonic acid content in SM from seeds of *urgt1-2* and *urgt2-2* mutants and rescue and promoter swap transgenic lines. SM was extracted by incubation with water, and Rha and GalA were quantified by HPAEC-PAD. GalA is shown along with Rha because they are the main components of RG-I in SM. Error bars represent SD from three biological replicates (*n* = 12). Statistical analyses were performed by using the Mann–Whitney test (*p* < 0.05).

To analyze whether the promoter swapping lines rescued the *urgt1-2* and *urgt2-2* phenotypes, we analyzed the monosaccharide content in SM and AIR from 6-week-old rosette leaves from WT and all the transgenic lines (Figure 6 and Supplementary Table 1). As we mentioned before, *urgt1-2* plants have a reduction in galactose in rosette leaves (Rautengarten et al., 2014; Figures 1, 6A). In addition, we observed a slight decrease in GalA (Supplementary Table 1). The analysis of *urgt1-2* rescued and the promoter swap _*pro*_URGT1:URGT2_*Swp*_ transgenic lines showed that changes observed in rosette leaves from *urgt1* mutants were recovered to WT levels (Figure 1 and Supplementary Table 1). No changes in monosaccharide composition were observed in SM from WT, *urgt1-2*, and all transgenic plants. Furthermore, to determine whether URGT1 can functionally replace URGT2 in seed coat cells, we analyzed the Rha and GalA contents in SM from WT, *urgt2-2*, and all transgenic lines. We observed that rescued lines (_*pro*_URGT2:URGT2_*Res*_) recovered the Rha and GalA contents in SM to WT levels. Interestingly, this recovery was also observed when *URGT1* is expressed in the *urgt2-2* background under the control of the *URGT2* promoter (_*pro*_URGT2:URGT1_*Swap*_) (Figure 6B). All the other transgenic lines showed similar contents of galactose, rhamnose, and galacturonic acid contents in comparison to WT in both tissues (Figure 6B and Supplementary Table 2). Interestingly, as observed previously (Parra-Rojas et al., 2019), *urgt2-2* showed an increase in xylose content in SM in comparison to WT Col-0 (Supplementary Table 2). Furthermore, the increase in xylose was also observed in the rosette leaves from *urgt2-2* plants (Supplementary Table 1). Both the rescued and promoter swapping lines restored the xylose increase observed in the *urgt2-2* mutants.

## Discussion

Nucleotide sugar transporters are Golgi transmembrane proteins that transport NSs synthesized in the cytosol to the Golgi lumen, where they are used by GTs to polymerize cell wall polysaccharides and glycoproteins (Reyes and Orellana, 2008; Temple et al., 2016). To date, several NSTs have been characterized *in vitro*; however, there is limited information about the function of these proteins *in vivo*. Arabidopsis NSTs have been grouped into six clades by protein sequence homology (Rautengarten et al., 2014; Ebert et al., 2015). Within these six clades, clade I, also named NST-KT, contains the UDP-Rha/UDP-Gal transporters (URGTs) and UDP-Xyl transporter (UXT) subfamilies (Rautengarten et al., 2014; Ebert et al., 2015). The six members of the URGT subfamily, URGT1–URGT6, are very similar among them and can transport UDP-Rha and UDP-Gal *in vitro* (Rautengarten et al., 2014). URGT1 and URGT2 are highly conserved, and they have similar affinities to transport UDP-Rha and UDP-Gal *in vitro* (Rautengarten et al., 2014). However, mutant analyses for both genes revealed distinct cell wall changes: *urgt1* mutants showed a slight reduction in Gal content in cell walls of 6-week-old rosette leaves, while *urgt2* mutants presented a reduction in Rha and GalA in the SM layer of the seed coat (Rautengarten et al., 2014; Parra-Rojas et al., 2019). Depletion of Gal in rosette leaves, as well as the reduction of Rha and GalA in SM, were not complete, suggesting that other members of the URGT gene family play a role in compensating for the lack of URGT1 and URGT2 in the mutants. This idea is supported by data extracted from the eFP Browser database (Supplementary Figure 5) that show that all *URGTs* are expressed in vegetative rosette leaves and the seed coat of the linear cotyledon seed developmental stage. Considering these results, we wondered if *urgt1* and *urgt2* chemotypes depend on their specific tissue expression and the protein context, given that different protein partners could participate in the synthesis of non-cellulosic polysaccharides. To evaluate this, we switched the expression between *URGT1* and *URGT2* by performing a promoter swapping experiment to understand if URGT1 and URGT2 could play similar roles *in vivo*. This approach has been widely used to study whether similar proteins play the same role *in vivo* (Muto et al., 2007). A similar promoter swap experiment of two xylosyltransferases, IRX10 and IRX10-L, revealed that IRX10 is more important during plant development than is IRX10-L (Wu et al., 2010), confirming that this approach can validate *in vivo* protein function and specificities. Therefore, to demonstrate if URGT2 could rescue the *urgt1-2* rosette leaf chemotype and URGT1 could rescue *urgt2*-2 SM changes, we characterized promoter swapping transgenic lines for both *urgt1-2* (_*pro*_URGT1:URGT2_*Swp*_) and *urgt2*-2 (_*pro*_URGT2:URGT1_*Swp*_) mutants. As controls, we obtained molecular rescued transgenic lines for *urgt1-2* using _*pro*_URGT1:URGT1_*Res*_ and for *urgt2-2* using _*pro*_URGT2:URGT2_*Res*_.

The qRT-PCR analyses revealed that *URGT1* was highly expressed in 6-week-old rosette leaves and, in lower amounts but similar to that in *URGT2*, in 8-DAP developing seeds as was previously reported (Rautengarten et al., 2014). *urgt1-2* exhibits a complete reduction of *URGT1* expression in 6-week-old rosette leaves and 8-DAP seeds. The monosaccharide composition of the AIR from rosette leaves of the *urgt1-2* mutant showed only a slight reduction in Gal, which suggests a reduction in pectic galactan side chains, as described previously (Rautengarten et al., 2014). Intriguingly, the reduction in Gal content was accompanied with a reduction in GalA content (Supplementary Table 1), a result that was not observed previously, probably due to the differences in the acid hydrolysis of matrix polysaccharides (Rautengarten et al., 2014). The overexpression of *URGT1* produced an increase in Gal and a reduction in GalA content in Arabidopsis rosette leaves (Rautengarten et al., 2014). These results suggest that changes in the expression of *URGT1* led to changes in the synthesis of non-cellulosic polymer synthesis. As we expected, the expression of *URGT1* in _*pro*_URGT1:URGT1_*Res*_ lines was similar to WT levels, and these lines also recovered the reduction in Gal and GalA content in 6-week-old rosette leaves, confirming that this chemotype is due to the lack of *URGT1* expression (Figure 6 and Supplementary Table 1). The promoter swap transgenic lines (_*pro*_URGT1:URGT2_*Swp*_) successfully expressed *URGT2* in 6-week-old rosette leaves, whereas the *URGT1* expression levels remained almost depleted. The _*pro*_URGT1:URGT2_*Swp*_ lines also showed a recovery in the Gal and GalA content of rosette leaf cell walls, demonstrating that URGT2 can replace URGT1 and rescue the *urgt1-2* mutant phenotype *in vivo* (Figure 6).

It has been previously reported that *URGT2* has low expression levels in comparison to *URGT1* in almost all plant tissues throughout development, showing the highest expression levels in developing seeds (Rautengarten et al., 2014; Parra-Rojas et al., 2019). The analysis of *urgt2-2* plants revealed an almost complete reduction of *URGT2* expression in seeds and rosette leaves, whereas *URGT1* transcript levels in *urgt2-2* remained unaltered. As was previously described, the monosaccharide composition of *urgt2-2* showed a reduction on the total sugar content of SM due to a reduction of Rha and GalA monosaccharides, the main components of the RG-I polymer filling the mucilage pocket of Arabidopsis seeds (Rautengarten et al., 2014; Ralet et al., 2016; Parra-Rojas et al., 2019). The reduction in Rha and GalA monosaccharides was also accompanied by an increase of Xyl content, a result that was previously related to the polymerization of RG-I and the xylan ramifications presented in SM (Fabrissin et al., 2019; Parra-Rojas et al., 2019). The analysis of *urgt2* molecular rescued lines (_*pro*_URGT2:URGT2_*Res*_) revealed a recovery of *URGT2* expression to WT levels in both tissues. Furthermore, the expression of *URGT2* in _*pro*_URGT2:URGT2_*Res*_ lines also led to a recovery of the total sugar amount of SM due to the restoration of Rha, Gal, and Xyl contents to WT levels. These results confirmed that the changes observed in *urgt2-2* mutant were due to the lack of *URGT2* expression. Furthermore, the _*pro*_URGT2:URGT1_*Swp*_ lines recovered the WT content of rhamnose, galacturonic acid, and xylose. These data confirm that the expression of *URGT1* in *urgt2* promoter swapping lines (_*pro*_URGT2:URGT1_*Swp*_) can also rescue the SM composition changes observed in the *urgt2-2* mutant.

Here, we demonstrate that switching the expression of URGT1 for URGT2, and *vice versa*, in promoter swapping lines can successfully rescue the phenotypes observed in *urgt1-2* and *urgt2-2* mutants. Surprisingly, although *URGT1* transcript levels were very similar to *URGT2* in WT developing seeds, the *urgt1-2* mutants did not show any change in SM composition. One possible explanation is that, focusing on the seed tissue expression available on the eFP Browser (Supplementary Figure 4A; Le et al., 2010), we observe that *URGT2* is expressed specifically in the seed coat integument which contains the epidermal cells that synthesize the mucilage pocket and also that it is expressed during the stages (linear cotyledon) in which these cells are actively synthesizing the mucilage components (Winter et al., 2007; Bassel et al., 2008). In contrast to *URGT2*, *URGT1* is less expressed in the seed coat integument and does not present the peak of expression at the stage when mucilage is being synthesized; therefore, these data suggest that both proteins play different roles in development of seed integument, and it could explain the absence of a mucilage phenotype in the *urgt1-2* mutant line (Supplementary Figure 4A). Interestingly, the expression of *URGT1* under the control of the *URGT2* promoter in _*pro*_URGT2:URGT1_*Swp*_ could successfully recover the *urgt2-2* mucilage chemotype. All these observations confirm that the pattern and timing of *URGT2* gene expression is essential to confer its specific role in seed development in Arabidopsis.

We provided evidence that URGT1 and URGT2 are exchangeable *in vivo*; thus, this poses the question of why their differential expression leads to different phenotypes. There are several plausible explanations for this question. One of them is that the absence of these NSTs should lead to a reduced pool of UDP-Rha and/or UDP-Gal inside the Golgi that could affect the synthesis of certain polysaccharides. Another explanation could be that the *in vivo* function of different NSTs depends upon the presence of polysaccharide synthesis proteins expressed in the same group of cells, tissue, or organ, thus defining the function of a given NST and supporting the idea that timing and place of expression are critical to defining polysaccharide composition throughout development. This idea is supported by the *URGT1* and *URGT2* co-expression networks obtained from ATTED-II (Supplementary Figures 4B,C; Obayashi et al., 2018). One of the closely *URGT1*-co-expressed genes is the previously reported galactan β-1,4-galactosyltransferase (GALS1; Liwanag et al., 2012; Ebert et al., 2018), which could be a good candidate to participate with URGT1 in the synthesis of pectic galactans and could explain the galactan phenotype observed in *urgt1-2* mutant rosette leaves. Interestingly, *URGT2* is highly co-expressed with the uronic acid transporters *UUAT1* and *UUAT3* and also with the galacturonosyltransferase-like 5 (*GATL5*), genes that participate in the synthesis of RG-I polymers of Arabidopsis seed coat mucilage, explaining the *urgt2-2* mucilage phenotype (Kong et al., 2013; Saez-Aguayo et al., 2017; Parra-Rojas et al., 2019). This work provided evidence supporting the idea that the *in vivo* activity of NSTs that are not monospecific depends on the timing expression of these genes, the cellular context, and the partners that are co-expressed with a given NST.

## Data Availability Statement

The raw data supporting the conclusions of this article will be made available by the authors, without undue reservation.

## Author Contributions

AL-G, SS-A, and AO designed the research. JC-B, JP-R, VA-M, and PS-O performed the experiments. JC-B, AL-G, SS-A, and AO analyzed the data. AL-G, HS-G, SS-A, and AO wrote the manuscript. All authors contributed to the article and approved the submitted version.

## Conflict of Interest

The authors declare that the research was conducted in the absence of any commercial or financial relationships that could be construed as a potential conflict of interest.

## Footnotes

1 http://abrc.osu.edu/

2 http://www.Arabidopsis.org

3 https://www.ebi.ac.uk/Tools/msa/muscle/

4 http://www.bioinformatics.org/sms2/ident_sim.html
