# Supplementary material for: Functional Interchangeability of Nucleotide Sugar Transporters URGT1 and URGT2 Reveals That urgt1 and urgt2 Cell Wall Chemotypes Depend on Their Spatio-Temporal Expression
Source: Front Plant Sci. 2020 Dec 8;11:594544. doi: 10.3389/fpls.2020.594544 (PMC7752924; doi:10.3389/fpls.2020.594544)
Supplement: Supplementary file 1 [file Table_1.DOCX]

Supplementary Material

# Supplementary Figures

#
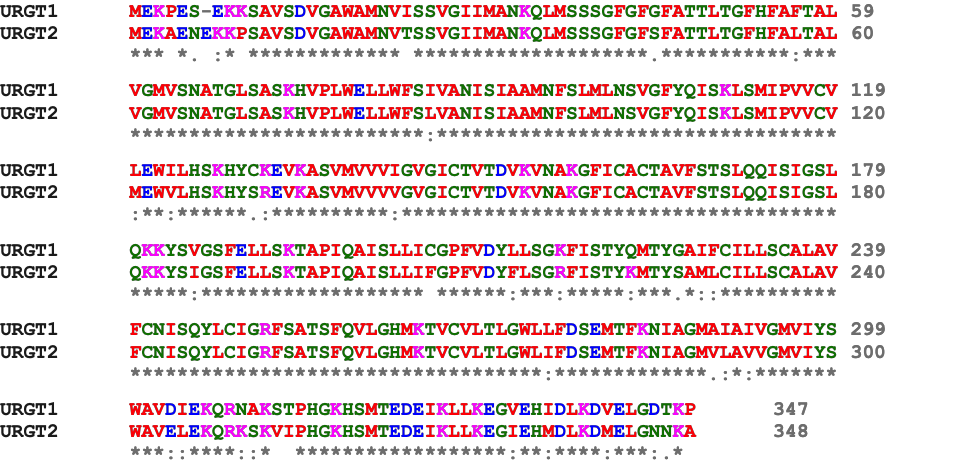


**Supplemental Figure S1. URGT1 and URGT2 protein alignment**

The alignment of URGT1 and URGT2 revealed that both proteins were highly conserved (89.4% identity and 93.4% similarity). URGT protein alignment was performed with MUSCLE webtool.


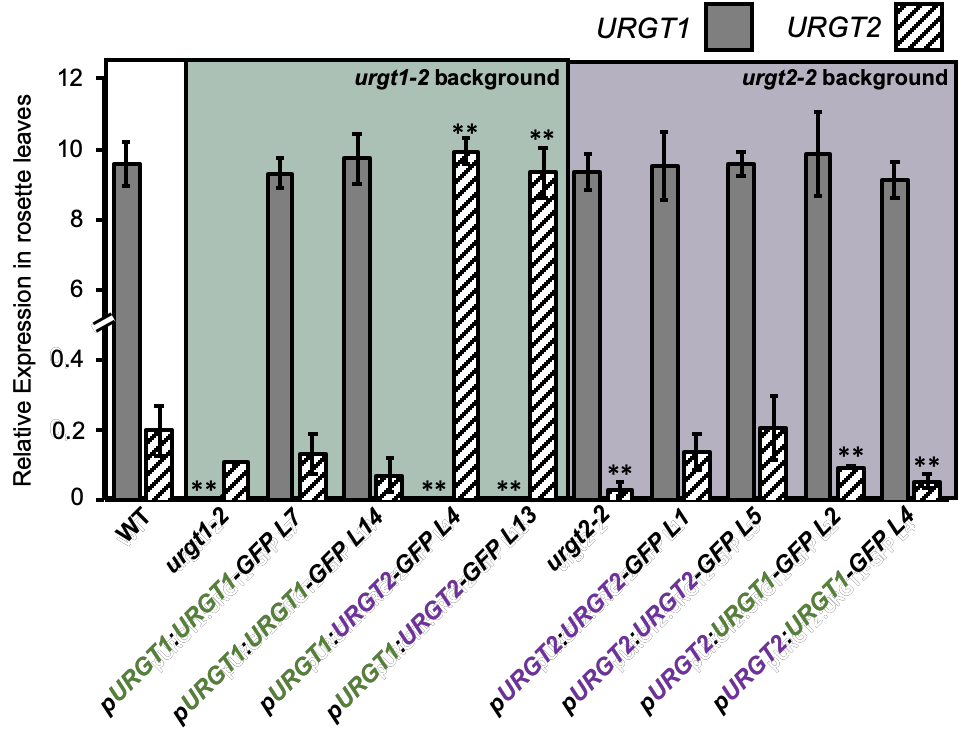


**Supplemental Figure S2. *URGT1* and *URGT2* expression in rosette leaves of *urgt1*, *urgt2* mutants, and two additional rescue and promoter swap transgenic lines**

q-RT-PCR analysis of *URGT1* and *URGT2* transcripts were performed from 6 weeks old rosette leaves from *urgt1-2*, *urgt2-2* mutants, two additional rescue lines (proURGT1:URGT1 L7 and L14 for *urgt1* mutant and proURGT2:URGT2 L1 and L5 for *urgt2* mutant) and two additional promoter swap transgenic lines (proURGT1:URGT2 L4 and L13 for *urgt1* mutant and proURGT2:URGT1 L2 and L4 for *urgt2* mutant). The values were calculated relative to *Clathrin* (*At5g46630*) and Ef1αA4 gene*.* Error bars represent SE values from 3 biological replicates (n=9). Statistical analyses were performed by using Mann Whitney test with **=p<0.001.


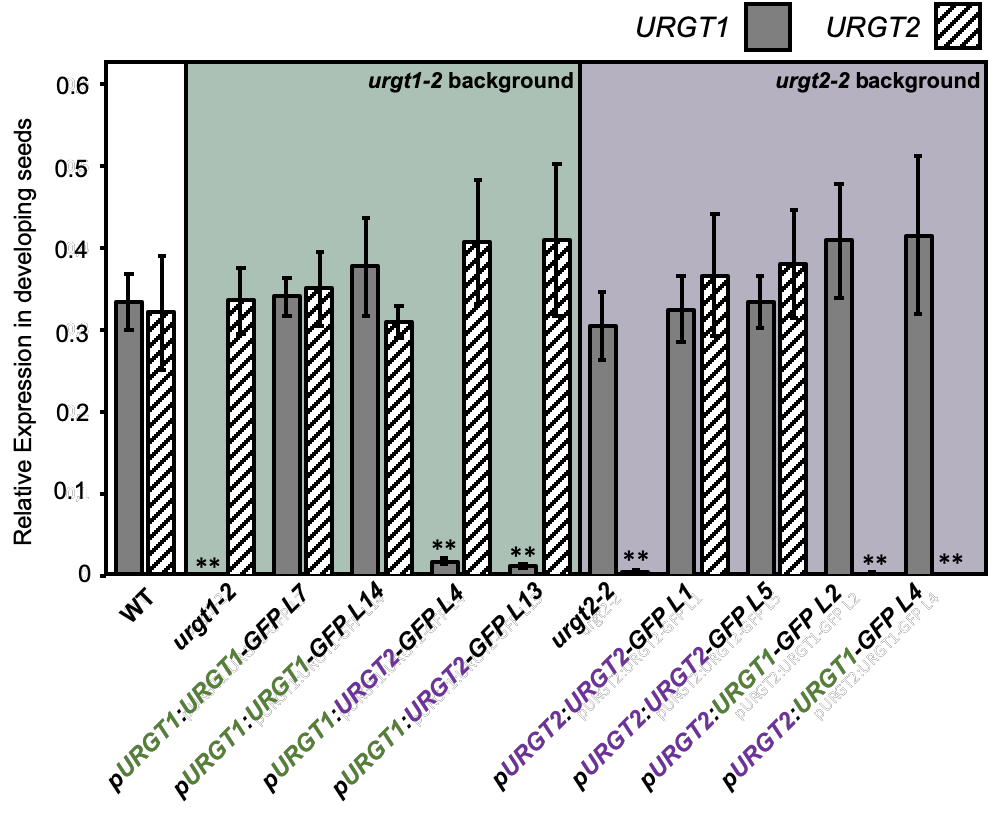


**Supplemental Figure S3: *URGT1* and *URGT2* expression in developing seed of *urgt1*, *urgt2* mutants, and two additional rescue lines and promoter swap transgenic lines**

q-RT-PCR analysis of *URGT1* and *URGT2* transcripts were performed from 8 DAP developing seeds from *urgt1-2*, *urgt2-2* mutants, two additional rescue lines (proURGT1:URGT1 L7 and L14 for *urgt1* mutant and proURGT2:URGT2 L1 and L5 for *urgt2* mutant) and two additional promoter swap transgenic lines (proURGT1:URGT2 L4 and L13 for *urgt1* mutant and proURGT2:URGT1 L2 and L4 for *urgt2* mutant). The values were calculated relative to seed specific reference gene (*At4g12590*) and Ef1αA4 gene*.* Error bars represent SE values from 3 biological replicates (n=9). Statistical analyses were performed by using Mann Whitney test with **=p<0.001

**
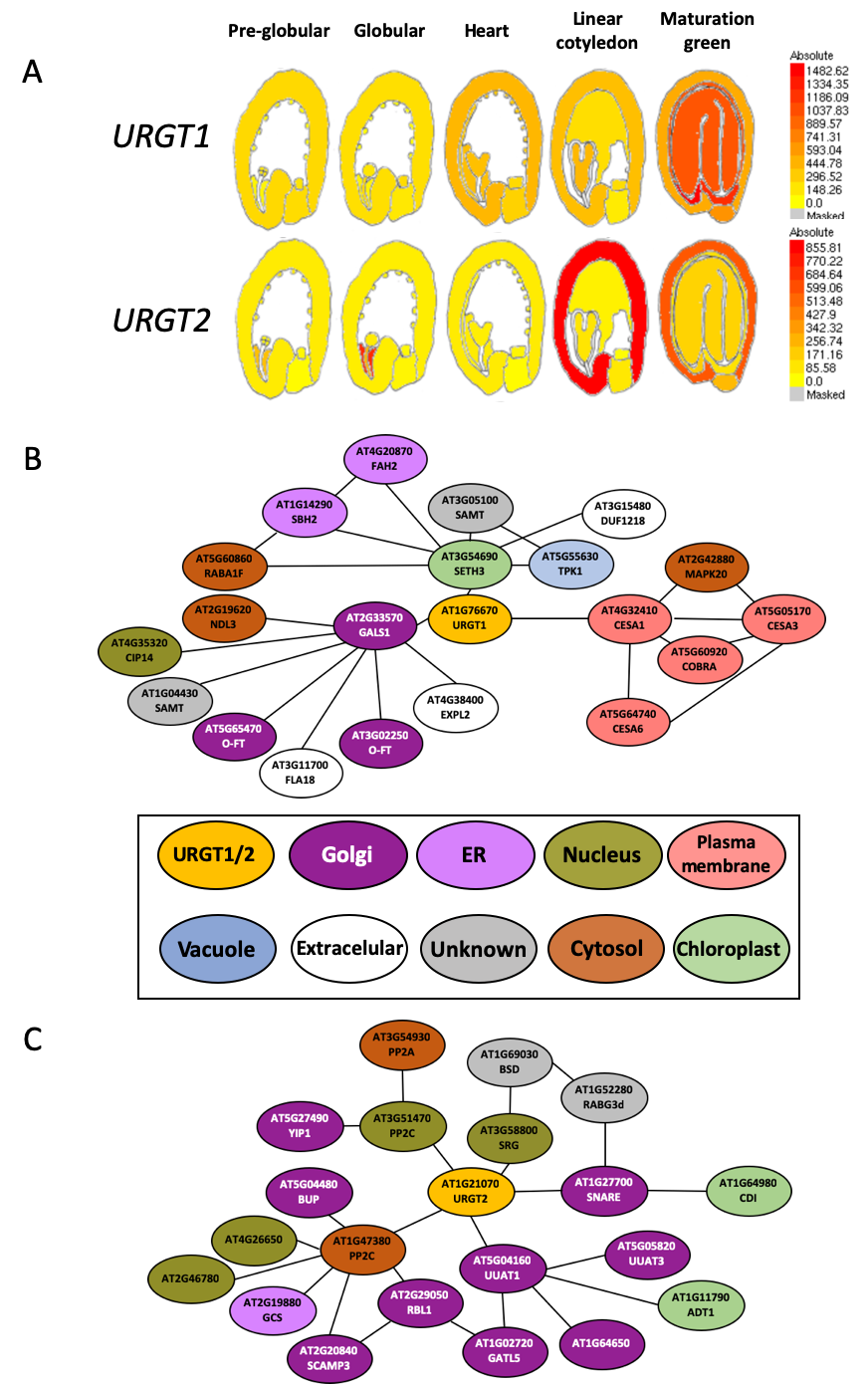
**

**Supplemental Figure S4: *URGT1* and *URGT2* gene expression during seed development and co-expression networks**

A. *URGT1* and *URGT2* expression in specific tissues during different stages of seed development. Gene expression was obtained from the Arabidopsis eFP Browser database (Winter et al., 2007; Le et al., 2010). *URGT1* is expressed in almost every tissue of the seed during its development, however *URGT2* is expressed exclusively in the seed coat integument.

B. *URGT1* co-expression network obtained from ATTED-II platform (Obayashi et al., 2018). The putative localization of the proteins was obtained from the Arabidopsis Information Resource (TAIR) (Lamesch et al., (2012). *GALS1*, Galactan synthase 1; *EXPL2*, Expansin-like 2; *O-FT*, O-fucosyltransferase; *FLA18*, Fasciclin like 18 AGP; *SAMT*, SAM methyltransferase; CIP14, CA interacting protein 14; NDL3, N-Myc downregulated-like 3; *SETH3*, D-Arabinose-5-P isomerase, *RABA1F*, *RAB* GTPase homolog A1F; *SBH2*, sphyngold base hydrolase 2; *FAH2*, Fatty acid hydrolase 2; *TPK1*, two-pore K+ channel 1; *DUF1218*, domain of unknown function 1218; *CESA1*, *CESA3* and *CESA6*, Cellulose synthase 1, 3 and 6; *MAPK20*, MAP kinase 20.

C. *URGT2* co-expression network obtained from ATTED-II platform (Obayashi et al., 2018). The putative localization of the proteins was obtained from the Arabidopsis Information Resource (TAIR) (Lamesch et al., (2012). *UUAT1* and *UUAT3*, Uronic acid transporter 1 and 3; *ADT1*, Arogenate dehydratase 1; *GATL5*, Galacturonosyltransferase-like 5, *RBL1*, Rhomboid-like 1; *SCAMP3*, Secretory carrier membrane protein 3; *BUP*, Bursting pollen; *PP2C*, protein phosphatase 2C; *YIP1*, Integral membrane Yip1; *PP2A*, protein phosphatase 2A; *SRG*, secretion-regulating guanidine nucleotide exchange factor; *BSD*, BSD domain protein; *RABG3d*, RAB GTPase homolog G3d; *SNARE*, syntaxin/t-SNARE protein; *CDI*, Cadmium induced protein.


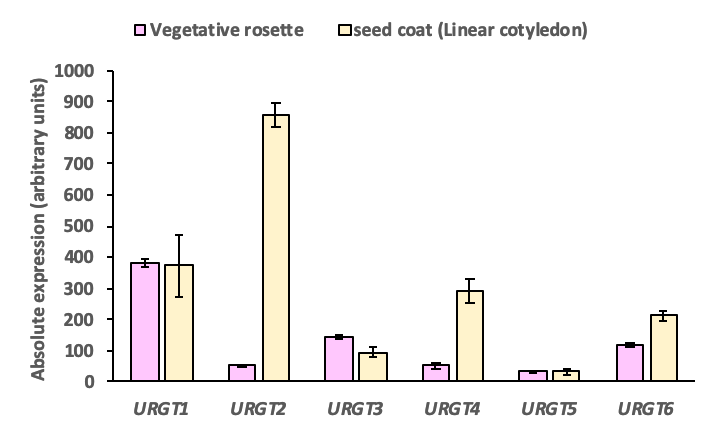


**Supplemental Figure S5. *URGT1-6* gene expression in vegetative rosette and in the seed coat of the linear cotyledon seed developmental stage.**

Absolute expression data was obtained from eFP Browser database (Winter et al., 2007; Le et al., 2010).

# Supplementary Tables

## Supplemental Table S1. Monosaccharide composition of AIR from rosette leaves of WT, *urgt1-2*, *urgt2-2*, rescue lines and promoter swap transgenic lines

| **Rosette leaves monosaccharides (mg of sugar/g of rosette AIR)** | | | | | | | | |
| --- | --- | --- | --- | --- | --- | --- | --- | --- |
|  |  |  | **pURGT1:URGT1-GFP** | | | **pURGT1:URGT2-GFP** | | |
| **Sugars** | **WT Col-0** | **urgt1** | **L7** | **L14** | **L82** | **L3** | **L4** | **L13** |
| **GalA** | 68.49 (3.20) | **55.34 (4.36)*** | **66.61 (4.85)** | **71.36 (5.54)** | **65.75 (5.66)** | **66.62 (5.07)** | **66.47 (4.35)** | **69.38 (5.18)** |
| **Rha** | 16.6 (1.20) | 15.68 (1.7) | 16.98 (1.69) | 17.65 (0.95) | 16.79 (2.15) | 16.47 (1.55) | 17.32 (1.31) | 17.91 (0.85) |
| **Fuc** | 4.80 (0.91) | 4.86 (1.1) | 5.07 (0.46) | 4.94 (0.50) | 5.35 (0.39) | 4.74 (0.41) | 4.59 (0.70) | 4.80 (0.20) |
| **Ara** | 15.38 (1.06) | 14.05 (1.74) | 16.18 (0.63) | 15.7 (1.27) | 16.58 (1.69) | 14.36 (2.55) | 15.96 (1.79) | 16.06 (0.99) |
| **Xyl** | 20.93 (1.28) | 19.45 (2.88) | 19.54 (3.09) | 20.8 (1.48) | 18.26 (1.27) | 19.66 (1.26) | 19.39 (4.10) | 18.91 (3.73) |
| **Man** | 7.27 (0.84) | 7.07 (0.96) | 7.58 (0.61) | 7.25 (0.58) | 7.17 (0.72) | 7.10 (1.09) | 7.17 (1.11) | 7.43 (0.81) |
| **Gal** | 20.8 (2.20) | **16.86 (1.05)*** | **22.07 (3.12)** | **21.36 (1.78)** | **22.36 (2.5)** | **21.69 (2.41)** | **19.85 (1.45)** | **21.13 (2.33)** |
| **GlcA** | 2.85 (0.52) | 2.30 (0.94) | 2.86 (0.54) | 2.78 (0.38) | 2.48 (0.76) | 2.51 (0.47) | 2.93 (0.48) | 2.49 (0.48) |
| **Total sugars** | 157.12 (7.02) | 135.61 (6.53)* | **156.89 (7.12)** | **161.84 (7.72)** | **154.74 (8.25)** | **153.15 (4.65)** | **153.68 (4.65)** | **158.11 (3.10)** |

| **Rosette leaves monosaccharides (mg of sugar/g of rosette AIR)** | | | | | | | | | |
| --- | --- | --- | --- | --- | --- | --- | --- | --- | --- |
|  |  |  | **pURGT2:URGT2-GFP** | | | **pURGT2:URGT1-GFP** | | | |
| **Sugars** | **WT Col-0** | **urgt2** | **L1** | **L4** | **L5** | **L2** | **L3** | **L4** |  |
| **GalA** | 68.49 (3.20) | 69.06 (4.84) | 64.55 (4.29) | 67.58 (4.17) | 66.97 (8.08) | 68.31 (2.90) | 66.29 (3.66) | 68.84 (3.08) |  |
| **Rha** | 16.60 (1.20) | 16.23 (0.69) | 17.60 (0.45) | 15.58 (1.74) | 17.56 (2.61) | 16.88 (1.18) | 16.88 (0.99) | 16.91 (0.70) |  |
| **Fuc** | 4.80 (0.91) | 4.90 (1.15) | 5.31 (0.49) | 4.77 (0.89) | 4.83 (0.84) | 4.73 (0.49) | 4.74 (0.25) | 4.60 (0.63) |  |
| **Ara** | 15.38 (1.06) | 14.74 (3.02) | 16.89 (2.77) | 14.19 (1.53) | 16.00 (2.38) | 13.01 (1.96) | 15.47 (1.27) | 14.22 (1.91) |  |
| **Xyl** | 20.93 (1.28) | **26.91 (1.03)*** | **19.07 (2.01)** | **21.14 (1.63)** | **19.18 (2.41)** | **18.01 (0.69)*** | **21.79 (2.42)** | **19.45 (1.96)** |  |
| **Man** | 7.27 (0.84) | 7.21 (0.76) | 7.56 (0.50) | 6.70 (1.19) | 7.10 (1.44) | 7.43 (0.73) | 7.65 (0.49) | 7.36 (0.51) |  |
| **Gal** | 20.80 (2.20) | 20.5 (3.15) | 21.34 (2.50) | 20.30 (2.42) | 20.63 (3.84) | 20.64 (2.13) | 21.77 (1.95) | 19.78 (2.22) |  |
| **GlcA** | 2.85 (0.52) | 2.77 (0.86) | 2.94 (0.58) | 2.18 (0.29) | 2.55 (0.35) | 2.77 (0.10) | 2.45 (0.57) | 2.53 (0.40) |  |
| **Total sugars** | 157.12 (7.02) | 162.32 (8.23) | 155.26 (9.09) | 152.44 (9.51) | 154.82 (16.12) | 151.78 (5.21) | 157.04 (5.11) | 153.69 (5.63) |  |

The monosaccharide composition of AIR extracted from 6 weeks old rosette leaves of all lines was measured using HPEAC-PAD. Means were calculated with data from three biological replicates and SDs are shown in parenthesis (n=12). Statistical analyses were performed by using Mann Whitney test (p<0.01). Asterisk represent significant differences of *urgt* mutant lines and the WT Col-0. Bold data showed differences between the transgenic lines and their respective background (*urgt1* or *urgt2*).

**Supplemental Table 2. Monosaccharide composition of soluble mucilage layer extracted from wild-type, *urgt1-2*, *urgt2-2*, recue lines and promoter-swap transgenic lines dry seeds**

| **Soluble mucilage monosaccharides (mg of sugar/g of dry seed)** | | | | | | | | |
| --- | --- | --- | --- | --- | --- | --- | --- | --- |
|  |  |  | **_pro_URGT1:URGT1_Res_** | | | **_pro_URGT1:URGT1_Swp_** | | |
| **Sugars** | **WT Col-0** | **urgt1** | **L7** | **L14** | **L82** | **L3** | **L4** | **L13** |
| **GalA** | 9.73 (0.74) | 10.18 (0.85) | 9.62 (0.62) | 9.53 (0.55) | 9.43 (0.71) | 9.53 (0.87) | 9.70 (0.77) | 9.94 (0.65) |
| **Rha** | 9.27 (0.61) | 8.82 (0.94) | 8.84 (0.62) | 9.20 (0.66) | 9.18 (0.39) | 8.88 (0.48) | 9.30 (0.55) | 9.55 (0.78) |
| **Fuc** | 0.10 (0.02) | 0.09 (0.03) | 0.11 (0.03) | 0.10 (0.02) | 0.10 (0.01) | 0.10 (0.01) | 0.10 (0.01) | 0.10 (0.03) |
| **Ara** | 0.21 (0.05) | 0.21 (0.04) | 0.20 (0.02) | 0.21 (0.04) | 0.20 (0.02) | 0.20 (0.02) | 0.21 (0.02) | 0.20 (0.01) |
| **Xyl** | 0.75 (0.07) | 0.78 (0.10) | 0.72 (0.07) | 0.76 (0.08) | 0.72 (0.08) | 0.74 (0.1) | 0.73 (0.08) | 0.81 (0.04) |
| **Man** | 0.20 (0.01) | 0.19 (0.03) | 0.19 (0.02) | 0.22 (0.05) | 0.20 (0.03) | 0.20 (0.01) | 0.20 (0.01) | 0.20 (0.03) |
| **Gal** | 0.46 (0.12) | 0.39 (0.11) | 0.40 (0.09) | 0.42 (0.05) | 0.44 (0.08) | 0.42 (0.07) | 0.41 (0.07) | 0.42 (0.06) |
| **GlcA** | 0.10 (0.04) | 0.11 (0.03) | 0.10 (0.05) | 0.10 (0.04) | 0.11 (0.03) | 0.11 (0.02) | 0.11 (0.04) | 0.11 (0.03) |
| **Total sugars (SM)** | 20.82 (1.87) | 20.77 (3.03) | 20.18 (1.32) | 20.54 (0.99) | 20.38 (0.19) | 20.18 (0.76) | 20.76 (0.14) | 21.33 (3.06) |

| **Soluble mucilage monosaccharides (mg of sugar/g of dry seed)** | | | | | | | | |
| --- | --- | --- | --- | --- | --- | --- | --- | --- |
|  |  |  | **_pro_URGT2:URGT2_Res_** | | | **_pro_URGT2:URGT1_Swp_** | | |
| **Sugars** | **WT Col-0** | **urgt2** | **L1** | **L4** | **L5** | **L2** | **L3** | **L4** |
| **GalA** | 9.73 (0.74) | **6.81 (0.83)*** | **9.22 (0.74)** | **9.17 (0.48)** | **9.3 (0.89)** | **8.75 (0.86)** | **8.9 (0.98)** | **8.99 (0.84)** |
| **Rha** | 9.27 (0.61) | **7.24 (0.55)*** | **9.04 (0.79)** | **8.61 (0.59)** | **8.83 (0.58)** | **8.24 (0.95)** | **8.41 (0.81)** | **8.57 (0.94)** |
| **Fuc** | 0.1 (0.02) | 0.1 (0.02) | 0.08 (0.03) | 0.08 (0.02) | 0.09 (0.01) | 0.1 (0.02) | 0.09 (0.03) | 0.09 (0.02) |
| **Ara** | 0.21 (0.05) | 0.2 (0.01) | 0.22 (0.04) | 0.2 (0.02) | 0.21 (0.03) | 0.21 (0.02) | 0.21 (0.01) | 0.2 (0.04) |
| **Xyl** | 0.75 (0.07) | **0.87 (0.09)*** | **0.74 (0.09)** | **0.74 (0.03)** | **0.77 (0.08)** | **0.77 (0.04)** | **0.71 (0.03)** | **0.76 (0.06)** |
| **Man** | 0.2 (0.01) | 0.19 (0.01) | 0.19 (0.08) | 0.19 (0.01) | 0.19 (0.03) | 0.22 (0.02) | 0.21 (0.02) | 0.19 (0.01) |
| **Gal** | 0.46 (0.12) | 0.39 (0.02) | 0.43 (0.08) | 0.41 (0.04) | 0.43 (0.07) | 0.43 (0.07) | 0.41 (0.09) | 0.4 (0.06) |
| **GlcA** | 0.1 (0.04) | 0.1 (0.01) | 0.09 (0.03) | 0.1 (0.04) | 0.12 (0.02) | 0.11 (0.04) | 0.09 (0.02) | 0.1 (0.03) |
| **Total sugars (SM)** | 20.82 (1.87) | 15.9 (1.68)* | **20.01 (2.26)** | **19.5 (1.9)** | **19.94 (1.54)** | **18.83 (1.41)** | **19.03 (0.34)** | **19.3 (1.34)** |

Soluble mucilage fractions (SM) were extracted by incubating mature dry seeds with water and their monosaccharide composition was obtained using HPEAC-PAD. Means were calculated with data from three biological replicates and SD were shown in parenthesis (n=12). Statistical analyses were performed by using Mann Whitney test (p<0.01). Asterisk represent significant differences of *urgt* mutant lines and the WT Col-0. Bold data showed differences between the transgenic lines and their respective background (*urgt1* or *urgt2*).
